# Decomposing Multifractal Crossovers

**DOI:** 10.3389/fphys.2017.00533

**Published:** 2017-07-26

**Authors:** Zoltan Nagy, Peter Mukli, Peter Herman, Andras Eke

**Affiliations:** ^1^Institute of Clinical Experimental Research, Semmelweis University Budapest, Hungary; ^2^Department of Physiology, Semmelweis University Budapest, Hungary; ^3^Department of Radiology and Biomedical Imaging, Yale University New Haven, CT, United States

**Keywords:** multifractality, focus-based multifractal analyses, multimodality, breakpoint, crossover, NIRS, EEG, fMRI-BOLD

## Abstract

Physiological processes—such as, the brain's resting-state electrical activity or hemodynamic fluctuations—exhibit scale-free temporal structuring. However, impacts common in biological systems such as, noise, multiple signal generators, or filtering by transport function, result in multimodal scaling that cannot be reliably assessed by standard analytical tools that assume unimodal scaling. Here, we present two methods to identify breakpoints or crossovers in multimodal multifractal scaling functions. These methods incorporate the robust iterative fitting approach of the focus-based multifractal formalism (FMF). The first approach (moment-wise scaling range adaptivity) allows for a breakpoint-based adaptive treatment that analyzes segregated scale-invariant ranges. The second method (scaling function decomposition method, SFD) is a crossover-based design aimed at decomposing signal constituents from multimodal scaling functions resulting from signal addition or co-sampling, such as, contamination by uncorrelated fractals. We demonstrated that these methods could handle multimodal, mono- or multifractal, and exact or empirical signals alike. Their precision was numerically characterized on ideal signals, and a robust performance was demonstrated on exemplary empirical signals capturing resting-state brain dynamics by near infrared spectroscopy (NIRS), electroencephalography (EEG), and blood oxygen level-dependent functional magnetic resonance imaging (fMRI-BOLD). The NIRS and fMRI-BOLD low-frequency fluctuations were dominated by a multifractal component over an underlying biologically relevant random noise, thus forming a bimodal signal. The crossover between the EEG signal components was found at the boundary between the δ and θ bands, suggesting an independent generator for the multifractal δ rhythm. The robust implementation of the SFD method should be regarded as essential in the seamless processing of large volumes of bimodal fMRI-BOLD imaging data for the topology of multifractal metrics free of the masking effect of the underlying random noise.

## Introduction

Fractal and multifractal concepts focus on characterizing scale-free properties in terms of scaling exponents—such as, spectral index (β) or Hurst exponent (*H*; Mandelbrot, 1982; Eke et al., 2002, 2012; Mukli et al., 2015)—of ideal or empirical signals. The scaling is a global behavior in the case of monofractals and a local property in the case of multifractals, which requires a set of exponents to be obtained for characterization. Specifically—in addition to a range of methods operating in the frequency and time/frequency domains (Eke et al., 2002)—in the time domain, this is achieved by analyzing a range of statistical moments (−∞ < *q* < + ∞) of the signal. In the monofractal case, a single *q*th order moment (i.e., the variance at *q* = 2) suffices for capturing the global roughness, *H*. However, for multifractals, a range of statistical moment orders are needed to obtain the generalized Hurst exponent, *H*(*q*). Submitting *H*(*q*) to the multifractal formalism yields the Hölder exponent, *h*, reflecting the local roughness of the process, and then the multifractal spectrum, *D*(*h*), which is essentially analogous with a histogram of local fractality in the signal. Accordingly, *D*(*h*) captures the moment-wise distribution of the singularity strength of local roughness or multifractal scaling in the temporal process (Kantelhardt et al., 2002; Ihlen, 2012; Mukli et al., 2015). We recently demonstrated that standard moment-based multifractal analyses were susceptible to signal inhomogeneity leading to spurious estimates of the multifractal spectrum. We resolved this issue by developing focus-based multifractal formalism (FMF), which replaced the standard—essentially monofractal—analysis for *H*(*q*) by fitting an exact multifractal to the family of moment-wise scaling functions all at once by enforcing an expected value at signal length (termed *focus*) as a guiding reference in the fitting procedure (Mukli et al., 2015). FMF explicitly relied on a previous observation on the focus (Kantelhardt et al., 2002) and can be related to some earlier multifractal approaches (Struzik, 1999; Struzik and Siebes, 2002).

**Figure 1:**
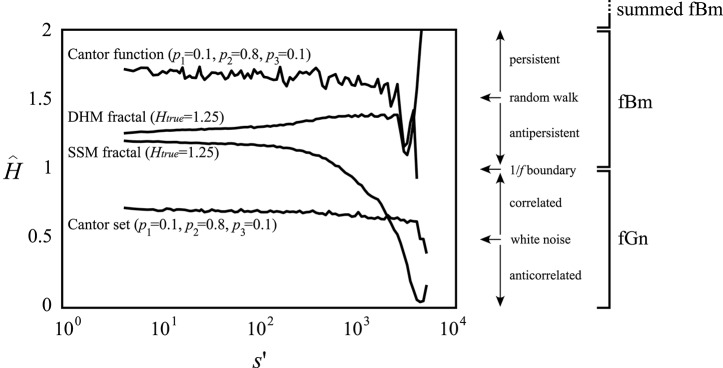
Scale-invariance cannot be revealed within inadequate scaling ranges. On synthesized monofractal signals of length *N* = 2^14^, the Hurst exponent as the measure of monofractality was estimated by the SSC method (Eke et al., 2000) within a temporal window with a lower boundary, *s*′, and an upper boundary set to *N*/2. The estimate of the generalized Hurst exponent at *q* = 2 [*H*(2)] was obtained on multifractal signals by the FMF-SSC method (Mukli et al., 2015) within the same range of scales. DHM fractal: synthesized by the method of Davies and Harte (DHM; Davies and Harte, 1987) at *H*_true_ = 1.25; SSM fractal: synthesized by the SSM method (Saupe, 1988); Cantor set and function generated at appropriate weight factors *p*_1_, *p*_2_, *p*_3_ (Cantor, 1883; Mandelbrot, 1982). Estimated Hurst and generalized Hurst exponents are displayed on an extended scale within the range of [0, 2]. Signal classes based on the extended *H* and *H*(2) are shown to the right for reference. Contracting scaling range—by increasing *s*′—will render the information remaining in the signal for its analysis inadequate to demonstrate its scale-invariance (see the bias in reference of *H*_true_ progressively increasing with *s*′). A standard treatment of scaling function multimodality typically results in contracted scaling ranges and thus results in a loss of valuable information on fractal scaling.

In the pure mathematical sense of the fractal concept, scaling should be present across an infinite range of scales; a property of ideal fractals with an exact generating algorithm (Mandelbrot, 1982), such as, Cantor set and function (Cantor, 1883). Fractality can be present in a statistical sense in sampled representations of temporal processes, as it is the case with fractional Gaussian noise (fGn) and Brownian motion (fBm; Mandelbrot and Van Ness, 1968; Eke et al., 2000, 2002). However, the estimation of fractality of even such exact fractal structures can become easily corrupted by the effect of sampling (see Figure 1), filters (Valencia et al., 2008), trends (Kantelhardt et al., 2001), shuffling (Kantelhardt et al., 2001), multiple fractal signal components (Thornton and Gilden, 2005), or other scale-dependent influences, resulting in multimodal scaling functions.

Many physical, natural, biological systems show multimodal, scale-invariant properties, for example, sunspot activities (Movahed et al., 2006), river water levels (Rego et al., 2013), human heartbeat time series (Peng et al., 1995; Gierałtowski et al., 2012), neuronal discharge dynamics (Blesic et al., 2003), human near infrared spectroscopy (NIRS) signals (Eke et al., 2006), local field potential (Bedard et al., 2006), human electroencephalography (EEG) signals (Gifani et al., 2007), rat blood oxygen level-dependent functional magnetic resonance imaging (fMRI-BOLD) data (Herman et al., 2011), human gait trajectories (Kuznetsov et al., 2013), and neonate diffuse optical tomography data (White et al., 2012). So far—among the numerous possible scale-dependent influences—only trends leading to artificial crossovers (Kantelhardt et al., 2001) have been specifically assessed within such multimodal signals. Common among these examples is that multimodality emerges, e.g., by superposition of two or more coexisting multifractal processes. Thermodynamic analogies of multifractality (Stanley and Meakin, 1988; Tel, 1988; Arneodo et al., 1995) have suggested that superpositioning could also lead to phase transition (Grassberger et al., 1988; Muzy et al., 1993; Arneodo et al., 1995; Radons and Stoop, 1996). Multimodality of the former case is seen in the initial step of multifractal formalism resulting in a *scale-dependent* impact on the scale-free pattern, while phase transition manifests itself at a later stage as a *moment-dependent* phenomenon captured as an inflected multiscale exponent, τ(*q*), at a critical *q*. This latter case has been detailed in the literature due to its demonstrated physiological relevance (Roux et al., 1999; Nicolay et al., 2007; Kestener et al., 2011); thus, our study focused on the former case of multimodality only.

**Figure 2:**
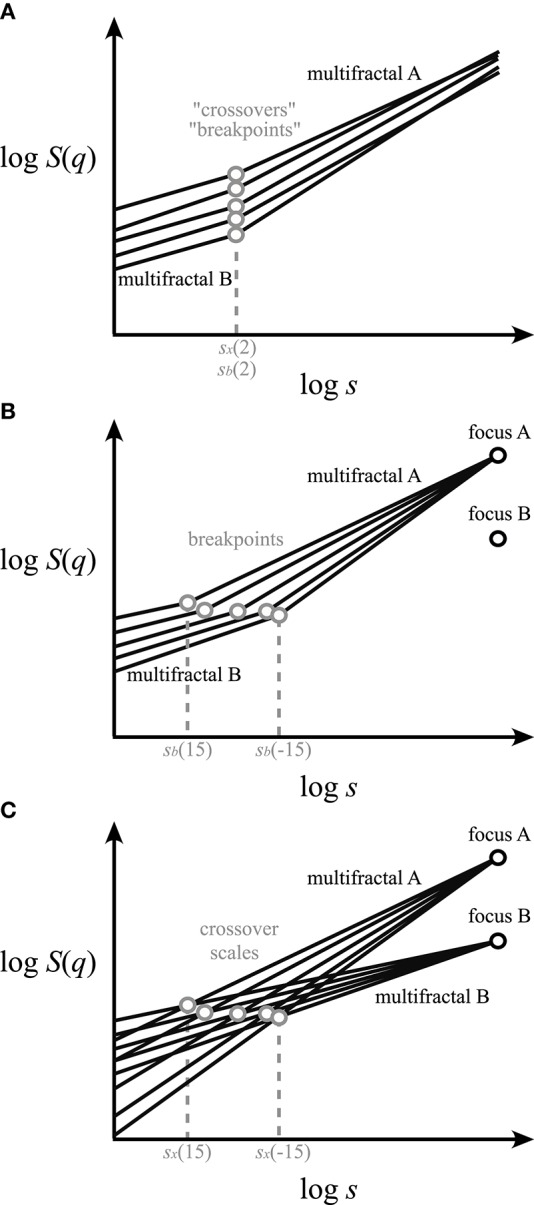
Different concepts for handling multifractal crossover. Exact scaling functions (solid lines) for a range of *q*s are shown in log-log plots. The components (multifractals **A,B**) separated by breakpoints—at the scale *s*_*b*_—and by crossovers—at the scale *s*_*x*_—are marked as gray circles. **(A)** Approaches in the literature for finding “crossovers” or “breakpoints” of bimodal multifractals along a single scaling function [i.e., log *S*(2)] should be regarded as vaguely defined (Schumann and Kantelhardt, 2011). **(B)** Our concept of *q*-wise scaling range adaptive focus-based multifractal formalism. This approach employs iterative fitting for *q*-dependent crossover scales by enforcing the focus (black circle) of the scaling functions found at maximum signal length. Note that the point-like *q*-wise breakpoints separate two adjacent SRs occupied by distinct scale-invariant components of the multifractal scaling functions. **(C)** Scaling function decomposition with focus-based multifractal formalism utilizing an extended version of the iterative approach shown in **(B)**. It yields a complete decomposition of the overlapping scaling functions of the merging fractals/multifractals coexisting within the same SR.

As characterization of multifractality in the time domain requires assessing moment-wise scaling exponents (Kantelhardt et al., 2001), multifractal multimodality should likewise also be described—along with the respective scaling exponents—in terms of moment-wise breakpoints or crossovers (Ludescher et al., 2011; Schumann and Kantelhardt, 2011; Gierałtowski et al., 2012; Figures 2B,C). In contrast, a mere presence of breakpoints or crossovers should by no means be taken as proof of multifractality (Matic et al., 2015).

Definition of “breakpoints” or “crossovers” appears inconsistent in the literature (Peng et al., 1995; Iyengar et al., 1996; Struzik et al., 1997; Kantelhardt et al., 2001; Ge and Leung, 2013; Kuznetsov et al., 2013; Rego et al., 2013), and their extension to multifractal multimodality (Figure 2A) is also lacking. In particular, recent works have either focused mostly on monofractal multimodality (Kuznetsov et al., 2013) or applied a monofractal approach to multimodal handling of multifractal signals (Ge and Leung, 2013; Rego et al., 2013; Figure 2A).

Previously, “breakpoints” or “crossovers” were determined by “eyeballing” or by segmented line regression (Ge and Leung, 2013) through scaling ranges (SRs). The latter being a typical adaptive analysis that identifies adjacent SRs with different scaling separated by a point-like breakpoint. As breakpoint is not a point-like attribute of the scaling function, some approaches omitted the transient in its vicinity (Kuznetsov et al., 2013) or applied fitting with multi-parametric models (Struzik et al., 1997). Admittedly, the benefit of these approaches is that they allow an approximation of fractal descriptors without formulating any a priori concept on signal genesis. Nevertheless, empirical signals much too often result from co-sampled signal processes, whose scaling may overlap across their respective breakpoints (Figure 2B); one such example is signal contamination by instrumental or biological noise. In these cases, the signal components obviously cross over around the apparent breakpoint (Figure 2C). This perception of multimodal signal genesis calls for an adequate concept for handling crossovers based on a genuine multifractal approach.

Accordingly, our aims were (i) to decompose the moment-wise crossover of superimposed multifractal signals based on an additive model, (ii) to validate this method, (iii) to compare this approach with an enhanced—moment-wise—version of the segmented line regression method, and (iv) to demonstrate their applicability on exemplary empirical signals.

## Materials and methods

Description of the methods of signal synthesis and empirical data acquisition are followed by introduction of two adaptive multifractal analyses of bi- or multimodal signals. The first approach is based on a *q*-wise identification of breakpoints along each and every scaling function as a step of signal pre-treatment, and hence is referred to as the *q*-wise scaling range adaptive (qSRA) method. The essence of the second method is to decompose the multifractal crossovers for all scaling functions of the analysis combined, thus achieving true scaling function decomposition (SFD) in a one-pass manner. As both apply to the regression scheme of our FMF (Mukli et al., 2015), these will be further referred to as the qSRA-FMF and SFD-FMF methods.

The multifractal algorithms, signal synthesis, and numerical tests were implemented in Matlab (The MathWorks, Inc., Natick, MA, USA) with code written by the authors. The multifractal toolbox containing scripts described in this paper can be requested from the corresponding author.

### Investigated signal populations

#### Synthesized monofractal time series

As described previously (Eke et al., 2000), statistically self-affine time series of the fGn type with exact long-range-dependent structuring were generated by the Davies and Harte method (DHM; Davies and Harte, 1987) at length, *N*, and pre-set Hurst exponent, ^fGn^*H*_true_. fBm-type time series were produced by cumulatively summing (Eke et al., 2000) their fGn counterparts, yielding a true extended Hurst exponent of *H*_true_ = ^fBm^*H*_true_ = ^fGn^*H*_true_ + 1. fBm-type time series were also produced directly at *N* and pre-set *H*_true_ = ^fBm^*H*_true_ by the spectral synthesis method (SSM; Saupe, 1988).

#### Synthesized multifractal time series

Cantor sets and—by their cumulative summation—Cantor functions as examples of exact multifractal structuring were generated at pre-set weight factors. Statistical self-similar multifractals with known *H*(*q*) were synthesized for testing purposes using the generalized binomial multifractal model (Oświȩcimka et al., 2006; Schumann and Kantelhardt, 2011). The generating algorithm is an iterative process in which a stochastic binomial cascade is created at a given weight factor and at a given length. In the generalized method, the degree of correlation and the strength of multifractality can be separately tuned, the former being set with the Fourier Filtering Method; for further details see Schumann and Kantelhardt (2011) and Mukli et al. (2015).

#### Synthesized multimodal time series

After considering various numerical testing frameworks, we chose the DHM algorithm and Cantor functions as offering the best control over the cardinal parameters in our testing. The above-listed signals represent cases of unimodality with a single SR. Multimodal synthetic (or mock) signals with multiple SRs were created by adding these unimodal fractal time series of known attributes (*N, H*_true_). Positioning of crossovers was controlled by setting the standard deviation (SD) ratios (i.e., focus ratio) of the signal components in addition to the differences in *H*_true_.

#### Sampled empirical time series

Human NIRS measurements using a NIRO 500 Cerebral Oxygen Monitor (Hamamatsu Photonics, Hersching, Germany) at a rate of 2 Hz were carried out to record the relative change in total hemoglobin concentration with a length of *N* = 16,384 data points (for details, see Eke et al., 2006). Human EEG signals were sampled with a length of *N* = 16,384 data points with eyes closed during random hand movements at 500 Hz using a Neurofax EEG System (see Acknowledgment; Pattnaik and Sarraf, in press). These empirical records were acquired from healthy volunteers above the pre-frontal area. Rat fMRI-BOLD data with a length of *N* = 4,096 data points were obtained by using a modified 11.7 T Bruker horizontal-bore spectrometer (Bruker AVANCE, Billerica, MA, USA) using a 1 H surface coil (1.4 cm diameter) with sequential sampling gradient echo planar imaging (EPI) sequence (Hyder et al., 1995): field of view of 2.56 × 2.56 cm^2^; image matrix of 64 × 64; slice thickness of 2 mm; repetition time of 200 ms (i.e., 5 Hz of sampling frequency) and echo time of 13 ms; and voxel size of 400 × 400 × 2000 μm^3^ (for further details see Herman et al., 2011).

### Multifractal analyses

According to its indirect concept, multifractal characterization of time series is performed by sequencing through the steps of scaling, regression, and singularity analyses of the multifractal formalism (Mukli et al., 2015). The output of the first step is the scaling function (*S*) obtained in the time domain [i.e., by DFA (detrended fluctuation analysis; Peng et al., 1994), SSC (signal summation conversion; Eke et al., 2000), or DMA (detrending moving average; Gu and Zhou, 2010)] and in the time-frequency domain (i.e., by WTMM (wavelet transform modulus maxima; Muzy et al., 1993) and WL (wavelet leader-based multifractal analysis methods; Jaffard, 2004). *S* is calculated from the input signal (*X*_*i*_) as a function of scale (*s*) and moment order (*q*) by obtaining the power mean of the measure (μ) with *q* as the exponent.

(1) $$\begin{array}{l} S[X_{i}](q,s)={\left(\frac{1}{N_{s}}\sum_{v=1}^{N_{s}}{\mu (v,s)}^{q}\right)}^{1/q}, \end{array}$$

where *N*_*s*_ stands for the number of non-overlapping windows, and *v* for different temporal positions within a particular signal segment of size *s*. For further details, see Kantelhardt et al. (2002) and Ihlen (2012).

Levels of moment order were selected from −15 to 15 in increments of 1, based on (i) the findings of Grech and Pamuła (2012) and (ii) the scaling parameter (Clauset et al., 2009) obtained from the thin-tailed distribution of our synthetic and empirical data. The scaling function of the signal was sampled in 100 logarithmically spaced scales, which is a much denser representation than dyadic sampling would be for a signal length *N* = 2^14^. Low and high temporal scales were excluded from our analyses (Cannon et al., 1997; Kantelhardt et al., 2002), resulting in a SR of 8 ≦ *s* ≦ *N*/2. The applied data were also tested by DFA with high-order detrending, effectively excluding an “artificial crossover” (Kantelhardt et al., 2001). We did not use filtering, as it produces a breakpoint effectively splitting the SR (Valencia et al., 2008). Disregarding this scenario (Matic et al., 2015) may well end up in the analysis of a filtering-ridden band for multifractality instead of the range of scale-invariance of the physiological signal.

#### Focus-based multifractal method

The FMF (Mukli et al., 2015) exploits the fact that the family of *q*-wise scaling functions yields a convergent structure merging at the coarsest scale, *s* = *N*. At this scale, the value defined in Equation (1) becomes *q*-independent yielding a “focus,” *S*[*X*_*i*_](*N*). Enforcing *S*[*X*_*i*_](*N*) as an analytical constraint improves the extraction of *q*-wise regression slopes for finite-length (by excluding inherently conflicting estimates due to the merely explicit emergence of this restriction) and thus offers a robust method for multifractal analysis of empirical data (Mukli et al., 2015; Ali et al., 2016; Delignières et al., 2016), the very approach adopted in this work. Instead of independently performing repetitive monofractal analyses on a set of empirical scaling functions (Equation 2), FMF methods—through minimizing the residual sum of squared errors, SSE—iteratively find the best-fitting, true multifractal and assign it to the family of the evaluated scaling functions (Equation 3).

(2) $$\begin{array}{l} SSE(q)=\sum_{s=s\min}^{s\max}(\left(\log s-\log x)\times \hat{H}[X_{i}](q)+\log\hat{S}[X_{i}](q,x\right)\\ \;{-\log S[X_{i}](q,s))}^{2}, \end{array}$$

(3) $$\begin{array}{l} SSE=\sum_{q=q\min}^{q\max}\sum_{s=s\min}^{s\max}(\left(\log s-\log N)\times \hat{H}[X_{i}](q\right)\\ \;{+\log\hat{S}[X_{i}](N)-\log S[X_{i}](q,s))}^{2}. \end{array}$$

In Equation (2), *x* has two specific values (at *x* = 0 and in the case of the “focus” *x* = *N*); otherwise, it represents the scale, where the exact scale-dependent statistic is being evaluated. The case of *x* = *N* and represents the enforced constraint in Equation (3). Thus, according to FMF, a set of model (i.e., exact) scaling functions with iterated parameters—Ĥ[*X*_*i*_](*q*) and log Ŝ[*X*_*i*_](*N*)—are fitted all at once to the actual data set of the scaling functions. In order to obtain an overall measure of the goodness-of-fit of the FMF regression procedure, its mean squared error (MSE) was calculated according to Equation (21) of Mukli et al. (2015).

#### Moment-wise scaling range adaptivity method

**Figure 3:**
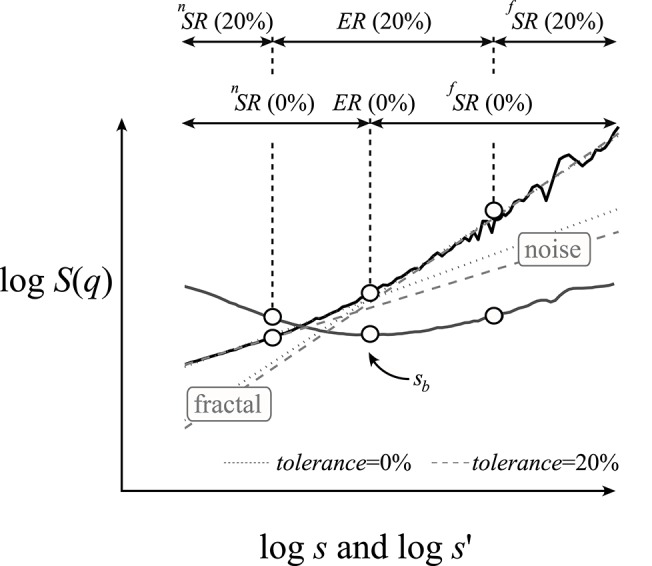
Numerical demonstration of the moment-wise scaling range adaptivity method. A bimodal, multifractal structure–function profile at *q* = 2 (solid black) was synthesized by DHM as the sum of fractal (*H*_true_ = 1.25) and noise (*H*_true_ = 0.5) signals with commensurable standard deviations. Regression slopes were determined by the DFA algorithm. The SSE(*q*,*s*′) function (solid gray line) at *q* = 2 is derived from Equation (3). The qSRA method finds the breakpoint (*s*_*b*_) at the minimum of this function. The exclusion range (ER, shown at a tolerance level of 20%) spans across scales where SSE(*q*,*s*′) < SSE_*tolerance*_ as calculated by Equation (5). In turn, the boundaries of the ER are set to the low and high edges of the adjacent scaling ranges for the underlying fractal and noise components, respectively. If *tolerance* = 0, then the ER is not excluded from the regression analysis (gray dashed regression lines). When *tolerance* = 0.2 (gray dotted regression lines), the estimated slopes better represent those of the underlying fractals. Other methods (such as, SSC) yielded isomorphic results (not shown).

The standard segmented line regression method is capable of finding breakpoints, *s*_*b*_, and also in the case of a superimposed signal (Figure 3) approximating crossovers, *s*_*x*_. Equation (4) is an adaptation of the segmented line regression method for a bimodal scaling function, where *s*′ could be any particular temporal scale. To capture *q*-dependent breakpoints, we introduced a *q*-wise regression algorithm, broken down into three steps of Equations (4a–c)

(4a) SnSE(q,s′)=∑s=smins′((logs−logx)×H^[nXi](q)                              +logS^[nXi](q,x)−logS[Xi](q,s))2,

(4b) SfSE(q,s′)=∑s=s′smax((logs−logx)×H^[fXi](q)                              +logS^[fXi](q,x)−logS[Xi](q,s))2,

(4c) $$\begin{array}{l} SSE(q,s\prime ){=}^{n}SSE(q,s\prime ){+}^{f}SSE(q,s\prime ), \end{array}$$

where indices ^*f*^ and ^*n*^ stand for different fractal processes: in our particular case uncorrelated (noise) and correlated (fractal) signals within a co-sampled arrangement, respectively. We chose noise and fractal signals as the constituents of a bimodal signal in describing our methods because this was the case for bimodal cerebral hemodynamic data reported earlier (Eke et al., 2006; Herman et al., 2011) and is used in this study as an exemplary dataset. The breakpoint for a given moment is obtained at the minimum value of the SSE(*q,s*′) function as the estimates, Ĥ[*X*_*i*_](*q*) and log Ŝ[*X*_*i*_](*q,x*) for the fractal and noise components, respectively, are being refined during the iteration process. Further away from the breakpoint, in the low range of scales, the underlying noise with low *H* will dominate, while in the high range of scales, the fractal component with high *H* will dominate. However, in between the two, where the respective function values are commensurable, their fluctuations blend into a non-fractal segment, a so-called exclusion range (ER; Figure 3). Consequently, by excluding the ER from the analysis, the error in assessing the low and high *H*s will be smaller, while for the FMF analysis yielding *H*(*q*), the error will increase due to SR being contracted by the exclusion range. To find the ER, we used Equation (5) (a similar approach as Equation (20) of Ge and Leung, 2013) at a specific *q* and selected a range of scales with SSE(*q*,*s*′) lower than SSE_*tolerance*_ calculated at a preset level of tolerance (0 ≤ *tolerance* ≤ 1)

(5) $$\begin{array}{l} SSE_{tolerance}(q)=\min SSE(q,s\prime )+tolerance\times (\max SSE(q,s\prime )\\ \;-\min SSE(q,s\prime )). \end{array}$$

Any moment-to-moment inconsistencies in the regression analysis will upset the expected structural aspect of multifractal scaling functions known as the “*H*(*q*)-monotonicity” (Mukli et al., 2015), the monotonous drop of regression slopes of *H*(*q*) from *q* = −∞ to *q* = +∞, because in subsequent steps of multifractal formalism this automatically results in “inversed” or “corrupted” multifractal spectra. To eliminate this eventuality, the analytical constraints of *H*[*X*_*i*_](*q*) > *H*[*X*_*i*_](*q*-*k*) with *k* > 0 were enforced on the nested iterative processes of minimization. MSE was obtained from the sum of SSEs from respective FMF analyses see Equation (3) on the noise and fractal components separated by *q*-wise breakpoints.

#### Scaling function decomposition method

**Figure 4:**
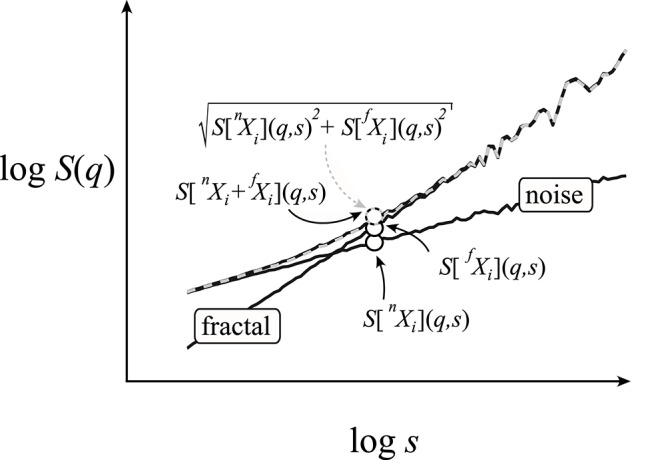
Numerical demonstration of the scaling function decomposition method. The two signal components (fractal and noise) of the bimodal signal are the same as shown in Figure 3. From these components, two bimodal signals were obtained: one by adding the raw signals (black) and the other their respective scaling functions (dashed gray). The three points represent exemplary values for this process at a given scale. The identical scaling functions demonstrate the validity of Equation (7) in the quantitative handling of bimodality—or for that matter—multimodality.

As fluctuations from the two underlying signal components mutually contribute to each other's scaling functions near the breakpoint, they hold estimates deviating from the power-law relationship (Figure 4). Taking the exemplary case of the SSC algorithm—where the statistical measure is the standard deviation, SD—this relationship is readily seen as a realization of the Bienaymé formula stating that in the case of uncorrelated variables, the variance (SD^2^) of their sum equals the sum of the respective variances (Bienaymé, 1853). A generalization for the case of (anti)correlated signals is given in the Appendix (See Supplementary Material). Thus, the emerging scaling function (the power mean of SD) can be well estimated (Figure A1 in Supplementary Material) as the root sum square of the composing scaling functions as

(6) S[Xi](q,s)=S[∑c=1NcXic](q,s)=∑c=1NcS[Xic](q,s)2,

where the signal (^*c*^*X*_*i*_) used in the calculation of *S* is in square brackets with *c* being a positive integer referring to each and every of the *N*_*c*_ constituent signals.

Earlier—for the cases of resting-state cerebral hemodynamic fluctuations—we showed that a fractally correlated signal is typically interwoven by uncorrelated noise (Eke et al., 2006; Herman et al., 2011). In this simplest case of bimodality, the above derivation can be reduced to the following direct approximation

(7) S[Xi](q,s)=S[Xin+fXi](q,s)≈S[nXi](q,s)2+S[fXi](q,s)2.

Accordingly, instead of fitting the two constituting fractally correlated components of a bimodal scaling function separately in two distinct processes, an exact bimodal model scaling function is reconstructed from two properly fitting power-law sets, based on the rule of addition Equation (7). Performing this one-pass regression on a log-log scale, the minimization of SSE—with the generalized Hurst exponent and the focus of the scaling function being iterated—results in the best fit of the exact bimodal model as follows

(8a) $$\begin{array}{l} \hat{S}{[}^{n}X_{i}](q,s)=\exp(\left(\log s-\log x)\times \hat{H}{[}^{n}X_{i}](q\right)\\ \;+\log\hat{S}{[}^{n}X_{i}](q,x)), \end{array}$$

(8b) $$\begin{array}{l} \hat{S}{[}^{f}X_{i}](q,s)=\exp(\left(\log s-\log x)\times \hat{H}{[}^{f}X_{i}](q\right)\\ \;+\log\hat{S}{[}^{f}X_{i}](q,x)), \end{array}$$

(8c) $$\begin{array}{l} SSE(q)=\sum_{s\;=s\min}^{s\max}(\log\sqrt{\hat{S}{[}^{n}X_{i}]{\left(q,s\right)}^{2}+\hat{S}{[}^{f}X_{i}]{\left(q,s\right)}^{2}}\\ {\;-\log S[X_{i}](q,s))}^{2}. \end{array}$$

A special application of this procedure is when one of the constituting components of the composite signal is known, which obviously reduces the number of tuning factors in the minimization process. Specifically, this component could be uncorrelated noise [i.e., instrument and/or biological noise (Peng et al., 1995; Blesic et al., 2003; Eke et al., 2006; Herman et al., 2011)] with a fractal estimate of Ĥ ≅ *H*_true_ = 0.5 [as will be seen later in Section Performance of the SFD Method on an Empirical Bimodal Signal with Limited Definition (fMRI-BOLD)].

SFD is not at all limited to *q*-wise applications, but can also be performed along with FMF. In this case, the process of minimization of the FMF analysis needs to be modified by raising the number of tuning parameters Equation (9). Thus, both the two sets (^*n*^*X*_*i*_ and ^*f*^*X*_*i*_) of *H*(*q*) and their associated two foci, *S*(*N*), see Equations (9a and 9b) are being simultaneously adjusted in the same iterative process Equation (9c)

(9a) $$\begin{array}{l} \hat{S}{[}^{n}X_{i}](q,s)=\exp(\left(\log s-\log N)\times \hat{H}{[}^{n}X_{i}](q\right)\\ \;+\log\hat{S}{[}^{n}X_{i}](N)), \end{array}$$

(9b) $$\begin{array}{l} \hat{S}{[}^{f}X_{i}](q,s)=\exp(\left(\log s-\log N)\times \hat{H}{[}^{f}X_{i}](q\right)\\ \;+\log\hat{S}{[}^{f}X_{i}](N)), \end{array}$$

(9c) $$\begin{array}{l} SSE=\sum_{q=q\min}^{q\max}\sum_{s=s\min}^{s\max}(\log\sqrt{\hat{S}{[}^{n}X_{i}]{\left(q,s\right)}^{2}+\hat{S}{[}^{f}X_{i}]{\left(q,s\right)}^{2}}\\ {\;-\log S[X_{i}](q,s))}^{2}. \end{array}$$

Similarly to the qSRA method, *H*(*q*)-monotonicity was granted by applying the same analytical constraints. The calculation of MSE from SSE Equation (9) was as explained in Section Focus-Based Multifractal Method.

The crossover scale, *s*_*x*_, of the decomposed scaling functions—where the respective statistical values are in principle the same—can be determined as the common value of the equations of the two underlying regression lines

(10) $$\begin{array}{l} \hat{H}{[}^{f}X_{i}](q)\times \log s_{x}(q)+\log\hat{S}{[}^{f}X_{i}](q,0)=\hat{H}{[}^{n}X_{i}](q)\\ \;\times \log s_{x}(q)+\log\hat{S}{[}^{n}X_{i}](q,0). \end{array}$$

Thus, the crossover scale can be calculated as

(11) $$\begin{array}{l} \log s_{x}(q)=\frac{\log\hat{S}{[}^{f}X_{i}](q,0)-\log\hat{S}{[}^{n}X_{i}](q,0)}{\hat{H}{[}^{n}X_{i}](q)-\hat{H}{[}^{f}X_{i}](q)}. \end{array}$$

When enforcing the respective foci of the underlying components, the best value of the crossover scale is obtained as

(12) $$\begin{array}{l} \log s_{x}(q)=\frac{\log\hat{S}{[}^{f}X_{i}](N)-\hat{H}{[}^{f}X_{i}](q)\times \log N-\log\hat{S}{[}^{n}X_{i}](N)+\hat{H}{[}^{n}X_{i}](q)\times \log N}{\hat{H}{[}^{n}X_{i}](q)-\hat{H}{[}^{f}X_{i}](q)}. \end{array}$$

### Characterization of methods

**Figure 5:**
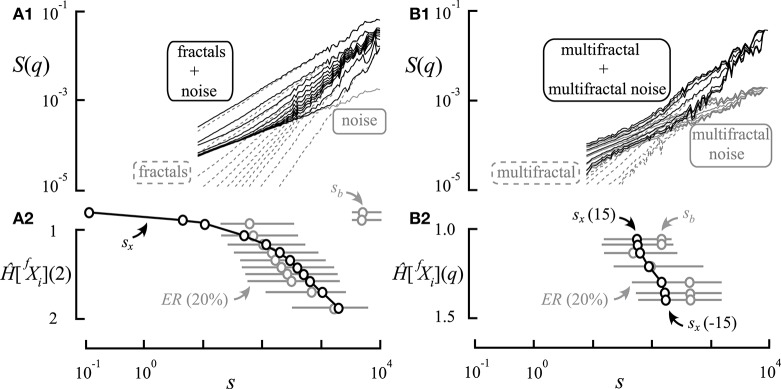
Impact of correlation and moment level on crossover scales. **(A1)** Twelve bimodal signals were generated by adding the scaling functions of 12 DHM-generated monofractal signals in length of 2^12^—representing varying degrees of correlation—and the same noise component of *H*_noise_ = 0.5. These signals were evaluated by scaling analysis for *S*(*q*)s. **(B1)** Scaling functions at seven moment levels of + 15 ≧ *q* ≧ −15 in increments of five are shown for the two constituents for demonstrating the use of signal addition in a multifractal setting. As seen **(A2)**, the breakpoints (gray circles), the exclusion ranges (gray bars), and the true crossover scales (black circles) become shifted toward larger scales with an increasing degree of correlation with the only exception being when *s*_*x*_ is occupying the lower scales. In this case, the algorithm will settle with a pseudo breakpoint at much larger scales where, due to increasing fluctuations, the first large enough hump in *S*(*q*) will be accidentally taken for a breakpoint (*s*_*b*_). When two multifractal components are merged, the analysis yields a similar distribution of breakpoints and crossover scales **(B2)** as determined by the actual span of *H*(*q*)s and the range of *q*s.

To assess the precision of our novel approaches in analyzing multifractal bimodal signals, estimates were compared with multifractal endpoints derived from the singularity spectrum, *D*(*h*), (Figure 5) and the results presented in the form of performance vignettes (Eke et al., 2012). These endpoints are *h*_max_ [the Hölder exponent, *h*, at the peak of *D*(*h*)] and *fwhm* [the full width of *D*(*h*) at half maximum] (Eke et al., 2012). *h*_max_ captures the degree of correlation, and *fwhm* can be taken as the measure of the supporting base of the singularity spectrum (see Figure 3 of Mukli et al., 2015).

For obtaining references for the estimates by subsequent SFD-FMF and qSRA-FMF methods, synthetic signal components were analyzed for their respective multifractal estimates with the FMF-DFA and FMF-SSC methods ((Mukli et al., 2015); Figure 5). The characterization of our methods was completed by testing their performance on empirical NIRS, EEG, and fMRI-BOLD signals.

## Results

### Impact of moment level on crossover scales

As seen in Figures 5A1,A2, the crossover between two components of markedly different correlation structuring is easy to detect. When *H* approaches *H*_noise_—as the true breakpoint becomes poorly defined—the bimodal signal approaches unimodal. A similar scenario is seen with the impact of moment level (Figures 5B1,B2), where the actual scale-wise distribution of crossovers will be determined by the dynamics of the *H*(*q*) of the signal components.

### Performance of qSRA and SFD methods on synthetic bimodal signals

**Figure 6:**
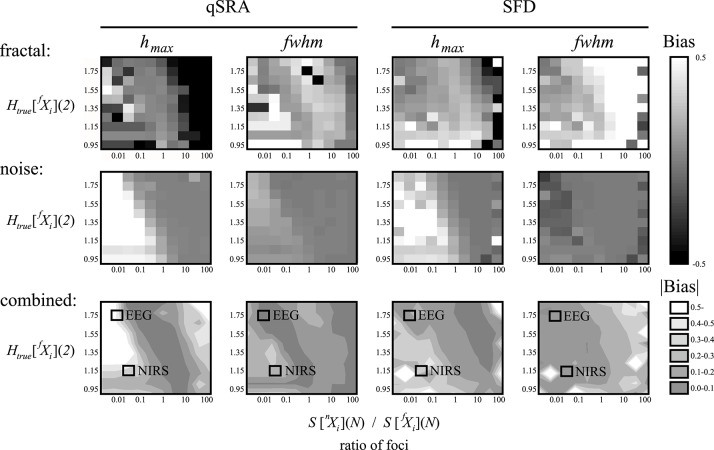
Performance of the qSRA and SFD methods on synthesized signals. A set of DHM-generated multifractal signals of length *N* = 2^12^ were created as a sum of fractal and noise components generated at *H*_true_[^*f*^*X*_*i*_] in steps of 0.1 and *H*_true_ [^*n*^*X*_*i*_] = 0.5 at pre-determined ratios of the respective foci. Values of correlation (*h*_max_) and multifractality (*fwhm*) were estimated for the *fractal* and *noise* components by qSRA- and SFD-FMF-SSC methods. Their biases with respect to estimates by FMF-SSC alone were plotted in intensity coded performance vignettes (Eke et al., 2012). The mean of the absolute biases for a *combined* evaluation of the fractal and noise components was also created and displayed in contour plots with the actual coordinates of the two empirical signals (EEG, NIRS) overlayed.

In addition to the impact of correlation and moment level, the focus has a decisive impact on how markedly a signal component dominates the bi- or multimodality of a composite signal (Figure 6). Accordingly, depending on the actual signal component, *H* and the component focus ratio (or *SD* ratio) together will impact the direction and magnitude of bias in the multifractal estimates (*h*_max_ and *fwhm*) for the two approaches alike. When the aim is to provide a characterization of multifractality for a bi- or multimodal multifractal signal (i.e., with *h*_max_ and *fwhm*, combined), the actual combination of *H* and the focus ratio should preferably be as close as possible to the diagonal band of low bias (Figure 6, combined).

An accurate multifractal output is at most partially qualified to assess performance. Determination of the proper method for a given signal is also a requirement. Lower MSE levels in the estimates of the SFD method when compared with those obtained by qSRA analysis suggests that the signals emerged as sums of two underlying scale-free processes, in which case the SFD method should be preferred. The performance of the SFD analysis was tested on the synthesized data pool used in Figure 6. The crossover-model, eventually identified by comparing the MSEs of our two methods (qSRA and SFD), showed a sensitivity of 73%.

### Performance of qSRA and SFD methods on high-definition empirical bimodal signals (EEG and NIRS)

**Figure 7:**
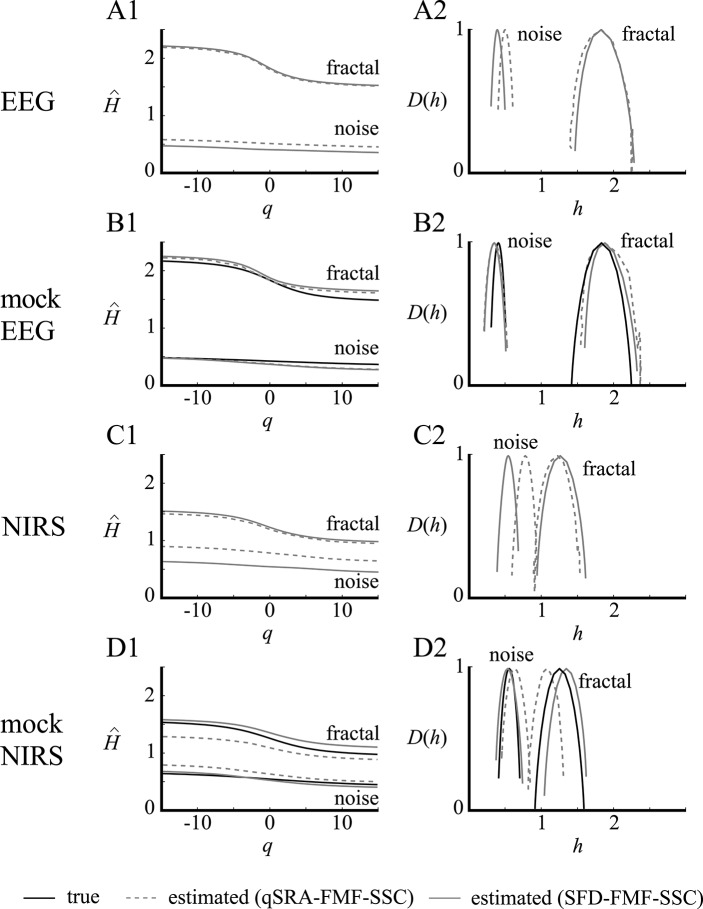
Performance of the qSRA and SFD approaches in handling multifractal bimodality on high-definition empirical signals (EEG and NIRS). EEG and NIRS signals recorded from the human brain were used as exemplary empirical signals in this demonstration. They were analyzed by qSRA- and SFD-FMF-SSC methods for *H*(*q*) and *D*(*h*) functions. Their synthetic equivalents (mocks) were created by adding fractal and noise components with foci, degree of correlations [*H*(2)], and multifractalities (Δ*H*_15_) matched to those of the empirical counterparts. As demonstrated by the closely matching true and estimated *H*(*q*) and *D*(*h*) functions for both the fractal and noise components of the mock signals, the SFD method proved clearly superior in handling the multifractal crossovers. Hence, the estimated *H*(*q*) and *D*(*h*) functions (A1, A2, C1, C2) should be regarded as realistic characterizations of the fractal and noise components of the bimodal empirical signals at the level of expectable bias shown in Figure 6.

High-definition empirical signals (EEG and NIRS in Figure 7) were chosen for demonstrating the optimal performance of the qSRA and SFD methods on empirical data. Both of these data sets had a combination of *H* and focus ratio close to the low-bias band of these methods (as seen in Figure 6, combined). The SFD method proved superior on these signals over the qSRA approach, yielding lower MSE-values and values of a magnitude lower when compared with those of the unimodal analysis (Table 1). Synthesizing the signal components based on the endpoint parameters of the SFD-FMF analysis (Table 2) yielded the same MSE when these components were added. This supports the notion that these bimodal signals could be treated as the sum of two concomitant processes, of which one could be fitted by an exact multifractal (Mukli et al., 2015) and the other by an exact multifractal noise (Grech and Pamuła, 2012). The multifractal analysis by the SFD FMF-SSC method for *H*(2) and *h*_max_ yielded a correlated level for the fractal and an uncorrelated level for the noise component. The multifractal measure (*fwhm*) was greater for the former than for the latter as shown in Table 2.

**Table 1:** The goodness-of-fit statistics (MSE) of the raw (FMF-SSC) and the two adaptive FMF-SSC methods (qSRA and SFD) for the empirical signals and their numerical equivalents shown in Figure 7.

**Method/Signal**	**EEG**	**mock EEG**	**NIRS**	**mock NIRS**
FMF-SSC	0.5226	0.5386	0.1388	0.2776
SRqA FMF-SSC	0.0353	0.0409	0.0388	0.0550
SFD FMF-SSC	0.0311	0.0320	0.0237	0.0260

**Table 2:** The endpoint parameters of SFD-FMF-SSC analysis of exemplary bimodal empirical signals shown in Figure 7.

**Method**	**Endpoint**	**EEG**		**NIRS**	
		**Noise**	**Fractal**	**Noise**	**Fractal**
SFD FMF-SSC	*h_max_*	0.41	1.83	0.55	1.26
	*fwhm*	0.18	0.66	0.24	0.54

**Figure 8:**
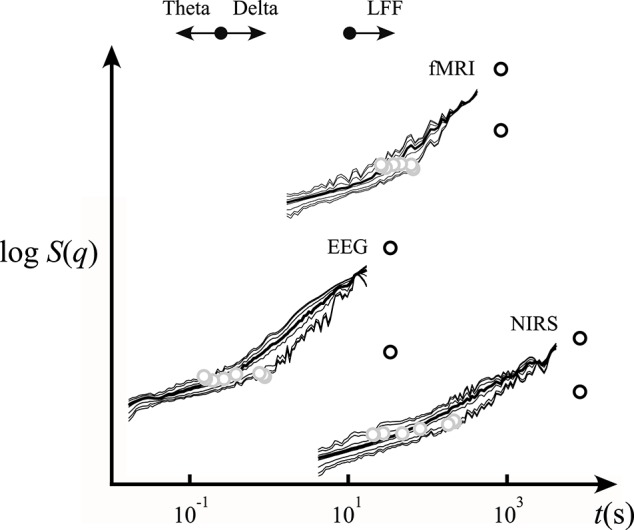
Scaling function representation of empirical signals interpreted by our SFD-FMF approach. The EEG and NIRS scaling functions were used in the analysis shown in Figure 7. For the properties of the EEG, NIRS, and fMRI signals see Section Methods. Shown are the respective scaling functions (solid lines), foci (black circles), and crossover scaling function values (gray circles). For further details, see the text.

The scaling functions for the EEG and NIRS data sets are shown in Figure 8. The estimated crossover scale of the human EEG is 257 ms at *q* = 2 and in case of NIRS records is 46 s at *q* = 2 (Figure 8). This demonstrates that the identified moment-wise crossover scales correspond well with characteristic boundaries between the theta and delta bands of the EEG and, in the case of NIRS signals, the transient is in-between the low- (Biswal et al., 2010; Herman et al., 2011) and high-frequency fluctuations (Figure 8). In this particular case, our analyses confirmed that bimodality in the analyzed EEG and NIRS signals (recorded from the human brain cortex) should not be regarded as a scale-dependent imprint of a transfer function but as one resulting from the superposition of random noise and correlated multifractal processes (Figures 7, 8 and Tables 1, 2).

### Performance of the SFD method on an empirical bimodal signal with limited definition (fMRI-BOLD)

**Figure 9:**
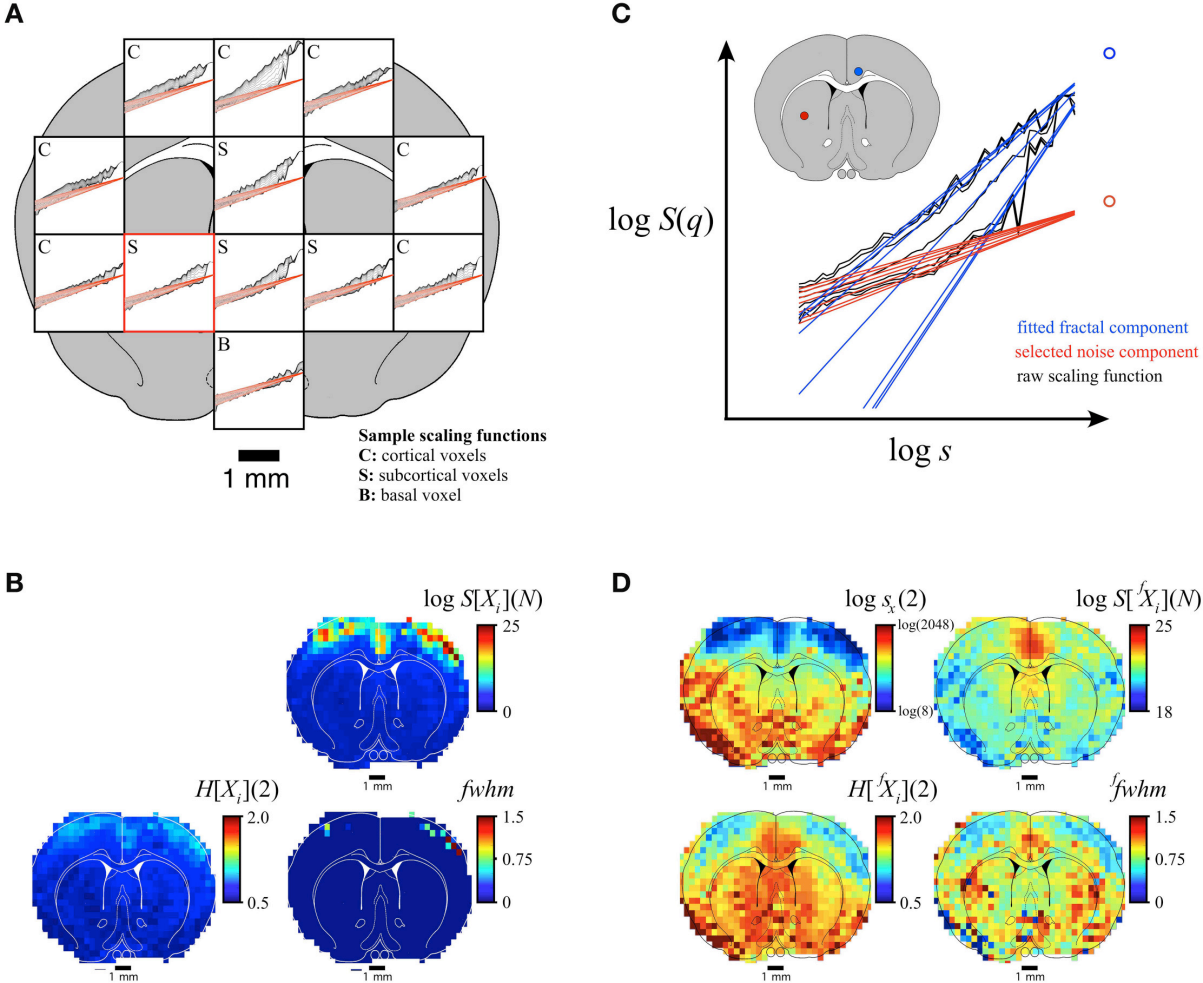
Performance of the SFD approach in handling multifractal bimodality on a limited-definition empirical signal (fMRI-BOLD). Results of voxel-wise analysis of rat fMRI-BOLD scan-based time series data (Herman et al., 2011) by the SFD-FMF method are shown along with those obtained by the FMF method for comparison. **(A)**: Representative scaling functions for cortical (C), subcortical (S), and basal (B) voxels are shown. The uncorrelated component determined from a voxel with mid-range *s*_*x*_ [marked in red in **(A,C)**] that fits very well as a ubiquitous noise component for all scaling functions. Standard, unimodal FMF-based analysis yielded the multifractal metric maps seen in **(B)**. The concept of the SFD-FMF-based adaptive, bimodal analysis is shown in **(C)**. The ubiquitous uncorrelated component (red) seen in **(A)** was used as default in decomposing the correlated (fractal) component for all scaling functions (see the representative black and blue functions with their associated foci). Parametric maps for the second moment crossover scale, Hurst exponent, and full-width-at-half-maximum of the multifractal singularity strength spectrum along with the focus of the generalized Hurst exponent function are seen in **(D)**. Note that only multimodal analysis can reveal the real topology of multifractality in the brain **(D)** that cannot possibly be captured by unimodal analysis **(B)**.

Rodent fMRI-BOLD imaging data of limited definition (Eke et al., 2012) was chosen to demonstrate the performance of our SFD method on scans of bimodal BOLD time series data obtained across a section at the level of the bregma (Herman et al., 2011; Figure 9A). Criteria of 0.5 < *H*[^*f*^*X*_*i*_](2) < 2, *s*_*x*_ within the full available SR, and 0.1 < *s*_*b*_/*s*_*x*_ < 10 yielded valid estimates of crossovers with MSE:SFD-FMF < MSE:qSRA-FMF at a rate of 68%. Accordingly, this assessment led to frequent dropouts in the parametric images at locations with extreme *s*_*x*_ (data not shown). We could, however, make the performance of the SFD method robust on limited-resolution BOLD data by exploiting the fact that a ubiquitous uncorrelated component with minor variations was present in the scaling functions throughout the section (Figure 9A) and thus could be used as a default in the analysis. Building on the maximal precision of the SFD method as demonstrated on synthetic signals (Figure 6), this default component was determined in a voxel with a mid-scale *s*_*x*_ (Figures 9A,C, marked in red). A reliable assessment of *s*_*x*_(*q*) resulted in consistent estimates of *H*(*q*) and *fwhm* as seen in the parametric maps in Figure 9D. Note that, in our case, because of the impact of the uncorrelated component, lower crossover scales appeared to be associated with lower Hurst exponent values in cortical areas with the exception of the cingulate cortex, where the correlated component was very dominant. This yielded apparently inverse patterns in the *H*[^*f*^*X*_*i*_](2) and *H*[*X*_*i*_](2) maps (Figures 9B,D). Furthermore, the crossover scale seen in the human NIRS signal (Figure 8) fell within the range of crossover scales found in the rodent fMRI-BOLD image data (Figure 9D).

## Discussion

We reported here on the SFD-FMF method as a genuinely multifractal approach to decompose the scale-free constituents of empirical bimodal signals by combining our multifractal formalism (Mukli et al., 2015) with the use of the Bienaymé formula (Bienaymé, 1853). We also developed qSRA-FMF, a moment- and FMF-based variant of the segmented line regression method (Ge and Leung, 2013) in order to obtain an MSE-based reference for discerning (i) additive from (ii) non-additive forms of signal genesis (see Figures 2A,B, respectively). Goodness-of-fit statistics—for given bimodal characteristics—were used as a guide in choosing between estimates obtained by the SFD-FMF or qSRA-FMF methods, as respectively valid. Accordingly, based on low MSE values, when analyzing bimodal EEG, NIRS, and fMRI-BOLD data we could demonstrate that these signals resulted from superposition. When applied to high-definition empirical signals (EEG, NIRS) with high degrees of freedom, these methods performed in a robust manner. Sub-optimally sampled physiological processes—such as, the exemplary fMRI-BOLD imaging data—however, obviously imposed limitations to the extent to which these methods could reveal bimodality in the signals; a circumstance that we could overcome by reducing the degrees of freedom in the analysis, thus yielding a robust performance on limited-definition fMRI-BOLD signals too.

### Physiological significance

Complex dynamics in biological systems—like that of the brain—have recently become the focus of intensive research as they represent an essential attribute for normal functioning (Bullmore et al., 2009). Staying with the example of the brain, multifractality is regarded as one of the main facets of complex, scale-free dynamics emerging from the underlying immense neuronal networks (Bullmore et al., 2009; Bullmore and Sporns, 2009). Multifractal characterization of scaling is inherently complex. It cannot be applied to empirical data without defining the application criteria in terms of the properties of the empirical signals in which resting-state brain dynamics are captured (i.e., EEG, MEG, NIRS, fMRI-BOLD; Eke et al., 2012). One such important property is multi- or bimodality that was the subject of this study.

The standard moment-based analyses of multifractal behavior, operating on the basis of an assumed unimodal model, estimates the scaling exponents within a single SR. This approach, however, will lead to erroneous estimates if unimodality does not hold. Indeed, it has been shown that EEG, NIRS, and fMRI-BOLD signals (Eke et al., 2006; Gifani et al., 2007; Herman et al., 2011) were in fact multimodal, a signal property that, therefore, must be taken into consideration in their multifractal characterization. To this end, our SFD method provides a means for decomposing the signal components of a bimodal signal with each part having its own set of single SRs needed to meet the aforementioned criteria for scale-invariance.

Beyond obtaining correct estimates for the scaling exponents, an understanding of the signal genesis in reference to the underlying physiological factors should be the subject of future research. Accordingly, in this work, we were motivated to develop the multifractal signal decomposition methods as needed and likely useful instruments to study multimodal signal genesis, in particular in the case of hemodynamic signals—such as, NIRS or fMRI-BOLD—that are widely used in brain connectivity research (Biswal et al., 2010; Mesquita et al., 2010). Both could be modeled as the convoluted product of ongoing regional neuronal activity (EEG) and the regional hemodynamic response function (HRF; Liu et al., 2011). As HRF is equivalent to low-pass filtering, it should render the hemodynamic signal multimodal with a breakpoint in its scaling function. Our qSRA and SFD methods can readily separate a breakpoint manifesting as signal convolution from a crossover resulting from signal superpositioning; we found the latter to be the case with the empirical signals studied. Despite its fundamental importance in signal genesis, this issue has not yet been addressed in the field of physiology or in particular in that of brain dynamics. Thus, our results on multifractal crossovers in the exemplary resting-state NIRS and fMRI-BOLD signals should be regarded as not only relevant but also promising in that our qSRA and SFD methods can reveal various facets of hemodynamic signal genesis in the brain.

### Crossover scales

Our FMF formalism (Mukli et al., 2015) as implemented in SFD-FMF offers an explicit framework to deal with the crossover of empirical multifractals. Incorporating the focus in the regression scheme of the SFD-FMF method allows for a robust estimation of crossovers in empirical signals. Omitting the use of the focus in guiding the regression process of *H*(*q*)—as is the case with standard methods like MF-DFA (Kantelhardt et al., 2002; Ihlen, 2012)—would potentially upset the moment-wise order of their independently obtained regression slopes. Ensuring such order of regression slopes in *H*(*q*) is essential in meeting the application criteria of the Legendre transformation incorporated in the multifractal formalism (Frisch and Parisi, 1985; Halsey et al., 1986; Bacry et al., 1993) and thus preventing outcomes with inversed or corrupted singularity spectra (Mukli et al., 2015; Delignières et al., 2016). To this end, Gierałtowski et al. (2012) attempted to circumvent the use of the Legendre transformation, known to be an inherently critical step in the formalism, with the multiscale assessment of *H*(*q*) (termed multiscale multifractal analysis, MMA). Nevertheless, a “reversed” *H*(*q*,*s*)—i.e., *H* increasing with *q*—as shown in Figure 17 of Gierałtowski et al. (2012) does evidently fail in meeting the above-mentioned criterion for a monotonously declining *H*(*q*,*s*) with increasing *q*s. This also explains the artifacts in the singularity spectra seen in their Figure 6. While their interpretation of the change of the average slope of the scaling function profile at some scale, *s*, would not suffice for multifractal analysis complete with singularity spectrum, it can still approximate breakpoints and/or crossover scales, but without discerning these phenomena. Nevertheless, there are other effective approaches to circumvent the difficulties associated with the use of the Legendere in obtaining *D*(*h*) (Jensen et al., 1987; Chhabra et al., 1989).

#### Impact of component focus ratio and temporal correlation

Additive random or correlated noise readily upsets multifractal analysis as demonstrated by Ludescher et al. (2011). In particular, when random noise with increasing amplitudes is added to a multifractal signal, the *q*-wise scaling function obtained by standard tools such as, MF-DFA will exhibit crossovers gradually shifting to larger scales. Accordingly, *H*(*q*) evaluated within the range of chosen scales becomes dominated by that of the added noise component. While the aim of these authors was not to provide a solution for handling noise contamination, they made an important contribution demonstrating that added noise propagates across scales ultimately leading to spurious results in multifractal analysis.

As for the impact of the component focus ratio (Figure 6), from the above-mentioned geometrical properties of multifractal scaling functions and the relationships shown in Figure 5A2 it follows that the crossover scale is low when both *H* and the component focus ratio are low (Figure 6, vignettes in lower left corner). Conversely, it is high when both *H* and the component focus ratio are high (Figure 6, vignettes in upper right corner). In between these extremes, a diagonal band of low bias due to the impact of mid-range crossover scales in the data is seen (Figure 6, bottom row) where the presence of merging scale-free patterns can be statistically confirmed (Clauset et al., 2009). When—due to the actual representation of scaling in the empirical data—the analysis is not performed within this optimized range, the signal definition should be improved: crossover scales that are much too low require an increased sampling rate, those that are much too high call for a longer signal to be collected (Eke et al., 2002).

**Figure 10:**
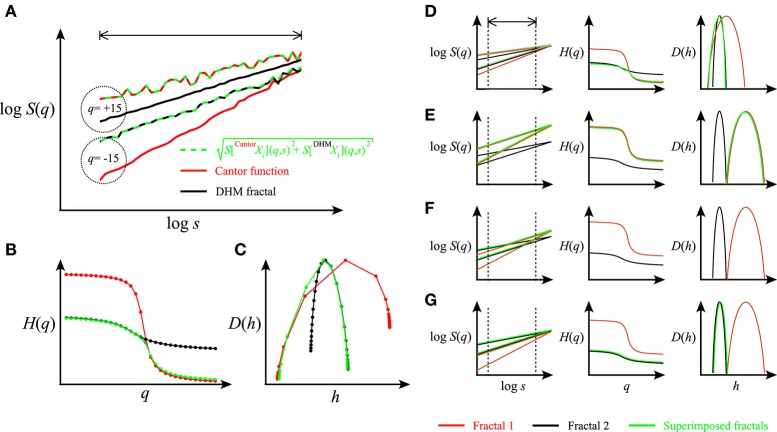
Representation of superimposed signal components in multifractal formalism. In **(A)**, scaling functions of a multifractal noise and a multifractal were generated by DHM (black) and Cantor function (red), respectively at moment levels of +15 and −15 with crossover (i.e., the intercept of scaling functions at identical *q* levels) falling outside the observed range of scales (indicated by the arrow bar). Their corresponding *H*(*q*) and *D*(*h*) are shown in **(C,D)**. The superimposed functions are indicated in green. Note that under the condition when the crossover falls outside the range of scales used in the analysis, the resulting *D*(*h*) becomes asymmetric in that the singularity strengths corresponding to multifractal noise and multifractal components end up being segregated in the negative and positive ranges of *q*, respectively. Hence, in this case the decomposition of the two signal components in *S*(*q*) for *H*(*q*) and *D*(*h*) across −15 ≤ *q* ≤ + 15 is not possible. In **(D–G)**, a collage is provided for component representation in *D*(*h*) for some typical cases depending on which of the components dominates the scale- and moment-wise dynamics. **(D)** Case of no crossover within the observed range of scales due to comparable foci and overlapping *H*(*q*) described in details in **(A–C)** note that this is the case of *q*-dependent phase transition where the dominance is *q*-wise, only resulting in a composite *D*(*h*) with no possibility of decomposition. **(E)** Case of no crossover and no composite *D*(*h*) due to the dominance of the multifractal. **(F)** Case of crossover with no dominance yielding decomposable *S*(*q*) and thus two separate *D*(*h*)s for the components. **(G)** Case of no crossover and no composite *D*(*h*) due to the dominance of the multifractal noise. Note that signal decomposition of the composite *S*(*q*) by our SFD approach is possible only in the case of F when crossover is present across the range of observation across a wide range of moment levels yielding a complete description of *H*(*q*) and *D*(*h*) of the components.

There are cases when the superposition of two fractal components yields a composite signal with a crossover falling outside the observed range of scales (Figure 10). The multifractal spectrum in this case is typically asymmetric (Drożdż and Oświęcimka, 2015). While under these conditions the crossover is not directly accessible to our SFD-based analysis, our additive model still allows for its characterization and offers an explanation for the asymmetry in *D*(*h*). This way, a composite process yielding asymmetric *D*(*h*) can be modeled too. Asymmetric *D*(*h*) can also be interpreted as a phase transition (Grassberger et al., 1988; Muzy et al., 1993; Arneodo et al., 1995; Radons and Stoop, 1996) based on the deep analogy that exists between the multifractal formalism and equilibrium statistical thermodynamics (Stanley and Meakin, 1988; Tel, 1988; Arneodo et al., 1995). According to this model, the superimposed partition functions under and above a critical *q* and the signals mutually perturb each other to a slight extent (Arneodo et al., 1995); thus, the partition function, τ(*q*) and *D*(*h*) are always dominated by a single component under which condition the Bienaymé formula yields similar results in describing the superposition ($S_{superimposed}\approx \sqrt{S_{component}^{2}+0}=S_{component}$).

#### Impact of moment level

In a scaling function representation of empirical temporal multifractality, the crossover scale for the chosen smallest negative moment is the largest and it becomes the smallest at the largest positive moment (see for example Figure 5B2). This moment-wise distribution of crossover scales emerges from the geometrical underpinnings of FMF (see Figure 2C) and the way *H*(*q*)-dependence is formulated in Equation (12) yielding the crossover scale itself. As crossover scales and breakpoints are similar manifestations of scaling, breakpoints should also be captured in a moment-wise manner.

The significance of the breakpoint in the analysis of bi- or multimodal signals has already been recognized in the literature (Peng et al., 1995; Kantelhardt et al., 2001; Eke et al., 2006; Herman et al., 2011; Ge and Leung, 2013). However, disregarding their moment-wise nature (Ge and Leung, 2013) distorts the acquired *H*(*q*)s with breakpoints falling outside the proper SR. This generates uneven error propagation across the moments in the case of standard multifractal methods (Ludescher et al., 2011), or introduces a slight but global error when FMF methods are applied. To the best of our knowledge, the qSRA- and SFD-FMF methods should be regarded as first attempts to carry out genuinely adaptive multifractal analyses on bi- or multimodal signals in a properly designed, moment-wise manner. They can readily be combined with various fractal and multifractal tools as their adaptive step, thus opening new possibilities for future applications.

### Significance of the fGn-fBm framework

**Figure 11:**
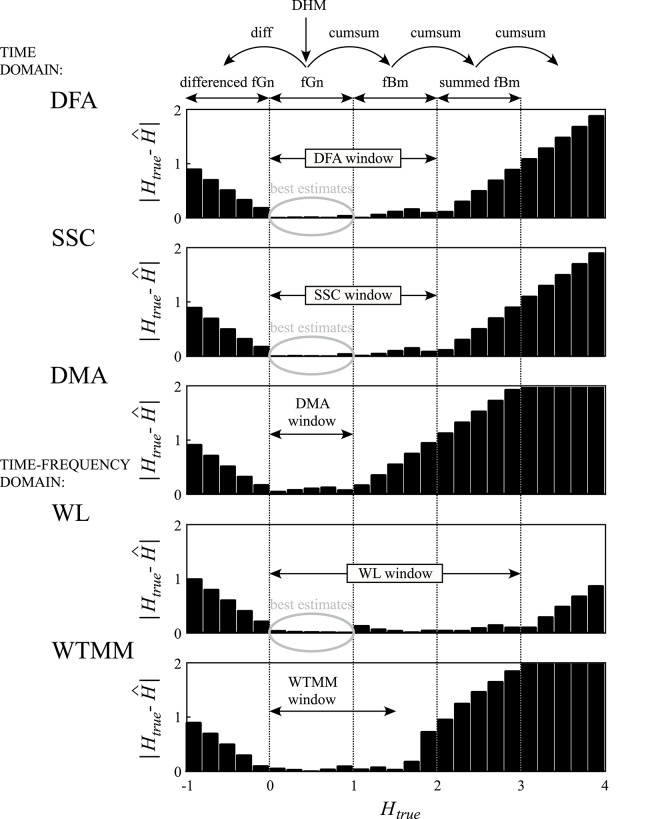
Performance of various fractal algorithms within the fGn-fBm framework on synthetic signals. Exact monofractal time series were generated by DHM for 0 < *H*_true_ < 1. Using the conversion rule of the framework, signals for −1< *H*_true_ < 4 were created by differencing (diff) and cumulative summation (cumsum) to obtain differenced fGn and summed fBm signals, respectively. Bias, as the absolute value of the difference of estimated and known *H*s, was trimmed to [0, 2]. Note that each of these methods has a range of *H*_true_ with minimal bias indicated by arrows and referred to as the *H*-window for the method. Above and below the *H*-window, estimates become increasingly biased due to saturation.

Mono- and multifractal analyses alike have been shown to benefit from the fGn-fBm fractal signal model of Mandelbrot and Van Ness (1968) as implemented by Eke et al. (2000). Despite its simplicity, this model captures the most fundamental properties of scale-free signals as realizations of fGn and fBm processes. These two signal classes are mutually convertible via cumulative summation or differencing of their subsequent values leading to an increase or decrease in their extended *H* by 1, respectively. As seen in Figure 11, methods differ in the range in which they yield minimal-bias estimates of extended *H* across the fGn-fBm framework. A multimodal signal may well contain components with *H* falling above or below the minimal-bias range. Hence, signal classification by methods such as, SSC (Eke et al., 2000) should be incorporated as a first step in the analysis. The actual implementation is by repetitive signal conversion until a difference in two successive *H* estimates is found of ~1. All of our signals and signal components proved one or other of the two classes of the fGn-fBm dichotomy. This way of handling signal classes in fractal analyses should reconcile issues seen with other adaptive approaches (Kuznetsov et al., 2013).

### Comparing overall performances and limitations of qSRA and SFD methods

The precision of the qSRA method increases with the level of tolerance, which in turn results in contracted SRs. This tends to weaken the estimates of *H*(*q*) due to falling short of securing wide enough scale-invariance (as seen in Figure 1). This effect is altogether eliminated by the SFD method, which makes use of all the data of the merging signal components.

**Figure 12:**
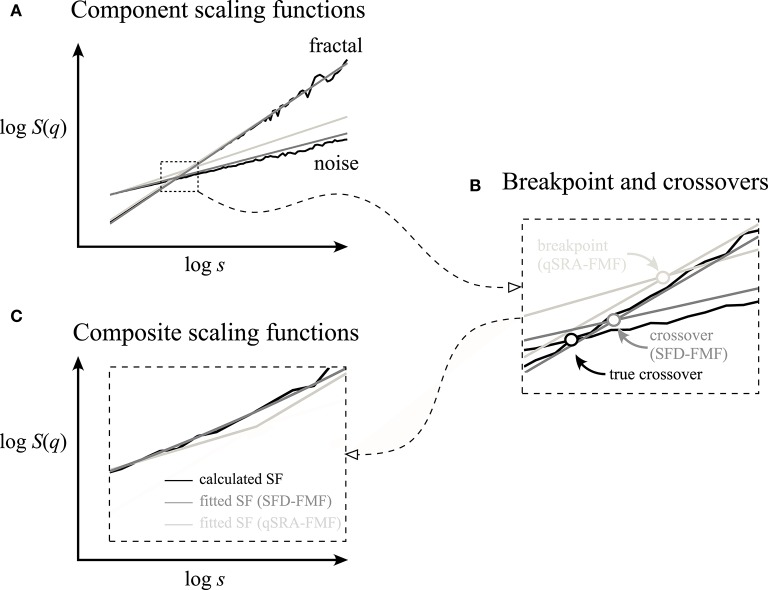
Breakpoints and crossover scales of superposition-type bimodality cannot possibly be identical. **(A)** Component scaling functions (fractal and noise) applied in Figures 3, 4 were used (solid black) to demonstrate the discrepancy in the underlying fractal components estimated by qSRA-FMF (light gray) and SFD-FMF (gray) methods. **(B)** The vicinity around the true crossover is shown enlarged. Note the difference between the true crossover scale and its estimate by SFD-FMF and the breakpoint estimated by qSRA-FMF. The former is due to the limited precision of the estimation by SFD-FMF, which both in principle and practice can be decreased. The latter cannot be minimized by improving the precision of qSRA-FMF owing to conceptual limitations preventing minimization of the difference between the true crossover scale and its estimation by a breakpoint. **(C)** Composite scaling functions were obtained by superpositioning the component time series (black line) or by applying the best fitted scheme of SFD-FMF and qSRA-FMF methods, respectively.

Our SFD method was validated against synthetic signals (Figures 6, 7, and Table 1). It outperformed the qSRA method for low-scale crossovers as the latter was shown to be susceptible to increased fluctuations typically seen in the large-scale region with limited number of available non-overlapping windows (Cannon et al., 1997). Hence, qSRA tends to locate the breakpoint for spuriously high scales (Figure 6). Multimodal scaling functions may deviate from the exact segmented line regression model near the breakpoint (Kuznetsov et al., 2013). This phenomenon always appears in superimposed fractals or multifractals, and can be modeled by added scaling functions (Figure 12).

### Significance of the design concept

The SFD approach is built around the notion that the multimodality emerges from the superposition of multiple and typically scale-free signal components. Multimodal multifractal scaling functions can also be produced by non-fractal generators like the infinitely divisible cascades (Chainais, 2007), filters, etc. Only specific generator models can adequately treat scale-dependent dynamics in the multimodal processes. As such, causal models are typically unavailable; our qSRA method can offer a phenomenological solution. Thus, where a causal generator model is sought, analysis by SFD should be preferred over that by qSRA. As the number of modeling parameters are the same in both cases, a smaller level of goodness-of-fit statistics (i.e., MSE, SSE) can indicate which of the two seems a realistic model for a particular signal (for a demonstration see Figures 7, 9). Performance of our methods and their discriminating power evidently depend on empirical signal definition (length, sampling, non-fractal contamination, etc.) as seen in the cases of our exemplary applications to high-definition EEG and NIRS, and limited-definition fMRI-BOLD signals.

### Performance of qSRA and SFD methods on empirical signals

#### Human EEG and NIRS signals

The crossover between the EEG signal components was found at the boundary between the δ and θ bands (Figure 8) of EEG classification. An independent δ and θ rhythm has already been proposed due to the significant interregional gap in synchrony (Mormann et al., 2008; Fetterhoff et al., 2015). Our current findings also prove the presence of an independent multifractal δ rhythm. The NIRS signal was also found to be the sum of temporally correlated, scale-free fluctuations dominated by low frequencies (i.e., low-frequency fluctuations) and uncorrelated [but not instrumental (Eke et al., 2006)] noise.

#### Rodent fMRI-BOLD imaging data

Resting-state brain dynamics as captured in fMRI-BOLD fluctuations is powered by ongoing neurodynamics spreading across the functional connections of a fractally organized anatomical network of an immense neuronal pool (Bullmore et al., 2009; Werner, 2010). Previously, we demonstrated that the resting-state fMRI-BOLD signal in an animal (Herman et al., 2011) and the fNIRS signal in a human model (Eke et al., 2006), alike, were bimodal temporal fractals. Thus, subject to future but likely advances in fMRI-BOLD technology, an improved signal definition may likely reveal bimodality in human fMRI-BOLD signals too. However, due to limitations inherent to the current fMRI-BOLD technology (Eke et al., 2012), their scale-free character—especially in cases of bimodality—is hard to capture (see Figure 12). Accordingly, while the SFD analysis yielded valid estimates of crossovers in a sizable fraction of the scanned voxels, still—depending on the correlation level and component focus ratio—numerous crossover estimates—due to sub-optimal data acquisition—proved invalid at extreme scales. This severely upset the topology of the multifractal parametric maps and prompted us to look for justifiable grounds to reduce the degree of freedom in finding breakpoints and/or crossovers in an attempt to improve the performance of the analysis. We did indeed find that the uncorrelated components of the scaling functions could be taken as default throughout the voxels of the scan. This uncorrelated component was determined at a site (Figure 9) where the precision of the method has been shown to be maximal in tests on synthetic signals (Figure 6). This procedure effectively compensated for the limited definition in the BOLD signals as seen in the enhanced topology of the parametric maps (Figure 9D).

## Conclusions and future perspectives

The issue of bimodality presents a major challenge when it comes to multifractal analysis of complex biological signals. We reported a novel approach (SFD-FMF method) as a genuinely multifractal tool to decompose the scale-free components of empirical bimodal signals by combining our multifractal formalism (Mukli et al., 2015) with the use of the Bienaymé formula (Bienaymé, 1853). We also developed a moment- and FMF-based variant of the segmented line regression algorithm (qSRA-FMF method) to discern additive from non-additive forms of signal genesis based on respective goodness-of-fit statistics. When applied to high-definition empirical signals (EEG, NIRS), these methods performed in a robust manner. The performance on sub-optimally sampled physiological signals is expected to be weaker; a circumstance that we could overcome by reducing the degree of freedom of the analysis, thus restoring a robust performance of the SFD-FMF and qSRA-FMF methods on a limited-definition fMRI-BOLD imaging dataset too. These methods offer the means to identify signal generators in physiological processes. Most importantly, they open ways to characterize the topology of key multifractal metrics in the brain emerging from its complex network dynamics.

## Author contributions

ZN developed the method and wrote the manuscript. PM performed numerical tests and analyzed empirical datasets for demonstration purposes. PH provided fMRI BOLD scans for demonstrational purposes. AE helped developing and writing the manuscript and provided conceptual guidance in the study.

### Conflict of interest statement

The authors declare that the research was conducted in the absence of any commercial or financial relationships that could be construed as a potential conflict of interest.

## Symbols and definitions

∧: estimated value
*A*: amplitude
β: spectral index
Δ*H*_15_: – the difference between the *H*(−15) and *H*(15) values
DFA: detrended fluctuation analysis
DHM: davies and harte method
DMA: detrending moving average
*D*(*h*): spectrum of singularity strength (or singularity spectrum for short)
EEG: electroencephalography
ER: exclusion range
*f*: frequency (Hz)
fBm: fractional Brownian motion (non-stationary signal)
fGn: fractional Gaussian noise (stationary signal)
FMF: focus-based multifractal formalism (an approach using a focus-based regression scheme)
fMRI: functional magnetic resonance imaging
fNIRS: functional near-infrared spectroscopy
^*f*^: measure describing the fractal signal component dominating over the higher scales
*fwhm*: full width of the singularity spectrum, *D*(*h*), at half of its maximum
*H*: extended Hurst exponent (fGn: 0<*H*<1; fBm: 1<*H*<2; summed fBm: 2<*H*<3)
*H*_true_: known value of the Hurst exponent in numerical syntheses of time series
*H*(*q*): generalized Hurst exponent
*h*: Hölder exponent
*h*_max_: the value of *h* at the peak position of *D*(*h*)
HRF: hemodynamic response function
*m*: detrending order (in DFA)
MF: multifractal (an approach using a standard regression scheme)
MSE: mean squared error
μ: measure
*N*: the length of time series (in data points)
^*n*^: measure describing the fractal (or noise) dominant over the lower scales
*N*_c_: number of constituent signals
NIRS: near-infrared spectroscopy
*N*_s_: number of non-overlapping segments
*q*: statistical moment order used in multifractal analysis (moment for short)
qSRA: moment-wise scaling range adaptivity (method)
*s*: temporal scale
*s*′: scaling boundary (possible breakpoint)
*s*_*b*_: breakpoint
*s*_*x*_: crossover scale
SD: standard deviation
*S*[*X*_*i*_](*q*,*s*): scaling function value at a given *q* and *s* calculated from signal *X*_*i*_
*S*[*X*_*i*_](*N*): the focus of the scaling function for signal *X*_*i*_
SR: scaling range
SFD: scaling function decomposition (method)
SSC: signal summation conversion (method)
SSE: sum of squared error
SSM: spectral synthesis method
*v*: the order of non-overlapping segments *v* = 1,…,*N*_s_
*x*: the scale selected for obtaining an actual value of a scaling function
*X*_*i*_: time series (signal), where *i* = 1,…,*N*
WL: wavelet leader (method)
WTMM: wavelet transfer modulus maxima (method)
