# Supplementary material for: Decomposing Multifractal Crossovers
Source: Front Physiol. 2017 Jul 26;8:533. doi: 10.3389/fphys.2017.00533 (PMC5527813; doi:10.3389/fphys.2017.00533)
Supplement: Supplementary file 1 [file Image1.pdf]

Appendix – Additive property of the scaling function

Variance of the sum of two variables (in our case signal *n* and *f*) is given as

$$Var[{}^{n}X_i + {}^{f}X_i] = Var[{}^{n}X_i] + Var[{}^{f}X_i] + 2 \times Cov[{}^{n}X_i, {}^{f}X_i] \,, \quad \text{(A1)}$$

where the covariance can be given with the help of *r*, the Pearson correlation coefficient as

$$Cov[{}^{n}X_i, {}^{f}X_i] = r[{}^{n}X_i, {}^{f}X_i] \times \sqrt{Var[{}^{n}X_i]} \times \sqrt{Var[{}^{f}X_i]} \,. \quad \text{(A2)}$$

The measure of the SSC algorithm is standard deviation, thus the square of its multiscaling structure (scaling function) is an averaged variance (as derived from Eq. 1) with corresponding properties. Due to the universal features of scaling functions, a generalization from the exemplary case of the SSC algorithm is possible, which results in a minimal moment-wise error (see Figure A1) (Mukli *et al.*, 2015). Therefore, substituting the squared scaling function and unifying Eq. A1 and Eq. A2 leads to the following moment (q)- and scale (s)-dependent formalism:

$$S[{}^{n}X_i + {}^{f}X_i](q,s) = \sqrt{S[{}^{n}X_i](q,s)^2 + S[{}^{f}X_i](q,s)^2 + 2 \times r[{}^{n}X_i, {}^{f}X_i](q,s) \times S[{}^{n}X_i](q,s) \times S[{}^{f}X_i](q,s)} \,. \quad \text{(A3)}$$

Expressing *r* from this equation yields a fractal-based, scale-wise correlation coefficient.

If the two signals are correlated at r=1, then based on Eq. A3 the scaling function of the superimposed correlated fractal yields the sum of two scaling functions

$$S[{}^{n}X_i + {}^{f}X_i](q,s) = S[{}^{n}X_i](q,s) + S[{}^{f}X_i](q,s) \,. \quad \text{(A4)}$$

If the two signals are anti-correlated at r=−1, than based on Eq. A3 the scaling function of the superimposed anti-correlated fractal will be the difference of two scaling functions

$$S[{}^{n}X_i + {}^{f}X_i](q,s) = S[{}^{n}X_i](q,s) - S[{}^{f}X_i](q,s) \,. \quad \text{(A5)}$$

If the two signals are uncorrelated at r=0, than based on Eq. A3 the scaling function of the superimposed uncorrelated fractal is the root sum square of the composing scaling functions (Bienaymé, 1853) (Eq. 7). As the constituent signals can typically be regarded as uncorrelated, decomposed fractals of different scale-invariance are best approximated with the latter approach.

References

Bienaymé, I.-J. (1853). Considérations à l'appui de la découverte de Laplace sur la loi de probabilité dans la méthode des moindres carrés. Crit. Rev. Acad. Sci. 37, 5-13.

Mukli, P., Nagy, Z., and Eke, A. (2015). Multifractal formalism by enforcing the universal behavior of scaling functions. Physica A 417, 150-167.

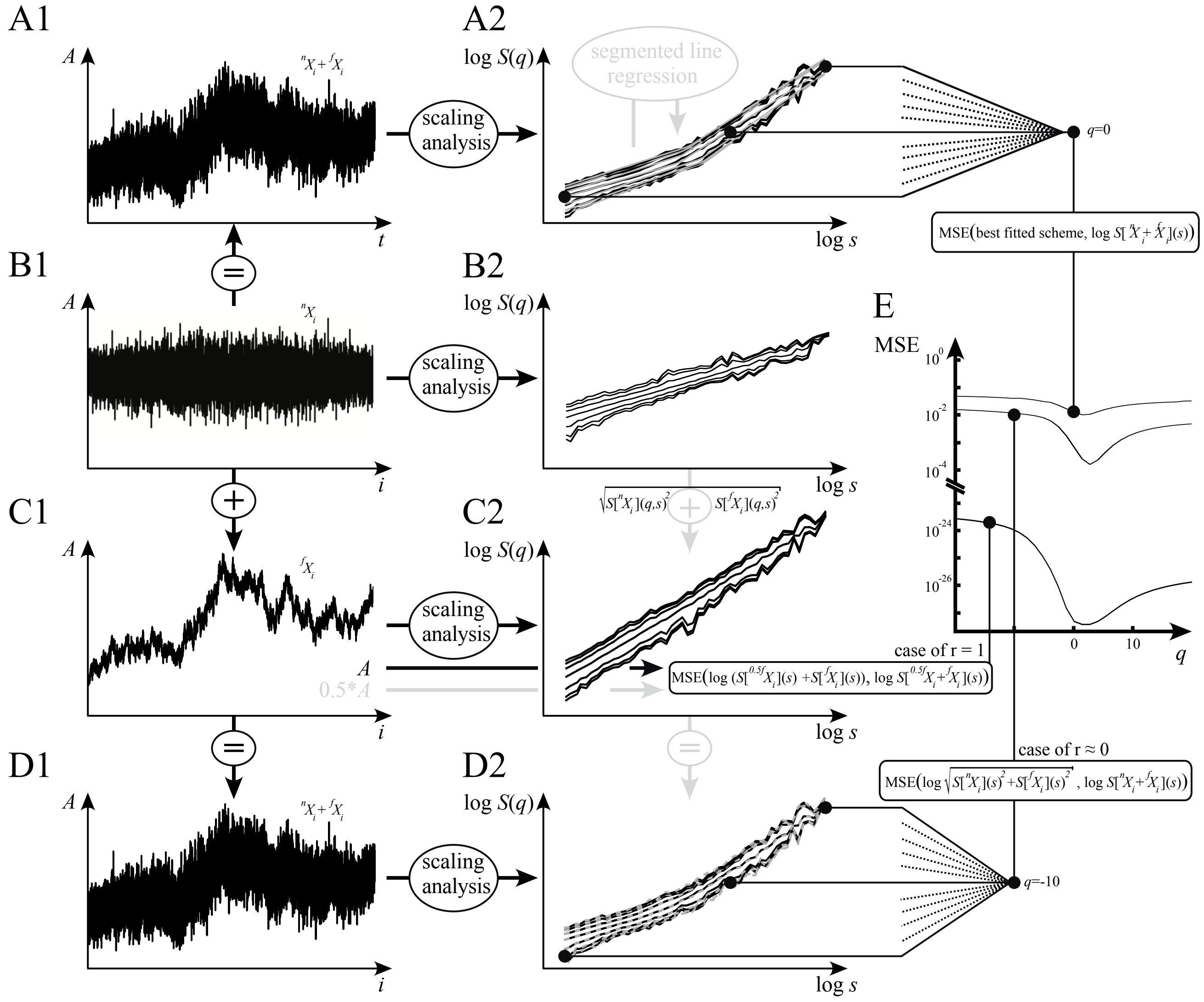

Figure A1. *Low-level moment-wise error validates the use of the Bienaymé formula in adding scaling functions of superimposed processes.* The noise and fractal components of the bimodal synthetic signals shown in Figures 3, 4, and 12 were used in this numeric simulation. They were generated by DHM at H_{true}=0.5 (B1) and H_{true}=1.25 (C1). Composite bimodal signals (A1=B1+C1 and D1=B1+C1) were obtained by adding the raw signals (superposition). All raw signals (A1, B1, C1, D1) were rendered to scaling function analysis for obtaining their respective scaling function profiles (black lines, A2, B2, C2, D2). Estimates obtained by the segmented line regression method are shown as a gray line (A2). Estimates by the Bienaymé formula are shown as a dashed gray line (D2). The effects of addition at the level of raw signals (D1=B1+C1) and at that of the scaling function (D2=B2+C2) were compared in terms of their respective moment-wise error, MSE(q) (E). Note that MSE(q) is the lowest for the case of adding two copies of the same raw signal differing only in their amplitudes, A, (C1+0.5*C1), which is identical to adding them at the SF level (C2+0.5*C2), both being the case of r=1. Addition by the Bienaymé formula applied for scaling functions (B2+C2=D2) (i.e., case of r=0) increases the level of MSE(q) as seen in panel E. Finally, MSE(q) is the highest in the case when the segmented line regression method is applied (A2→E); taken so far as the gold standard of bimodal fractal analysis.
